# CAGE sequencing reveals CFTR-dependent dysregulation of type I IFN signaling in activated cystic fibrosis macrophages

**DOI:** 10.1126/sciadv.adg5128

**Published:** 2023-05-26

**Authors:** Jonathan L. Gillan, Mithil Chokshi, Gareth R. Hardisty, Sara Clohisey Hendry, Daniel Prasca-Chamorro, Nicola J. Robinson, Benjamin Lasota, Richard Clark, Lee Murphy, Moira K. B. Whyte, J. Kenneth Baillie, Donald J. Davidson, Gang Bao, Robert D. Gray

**Affiliations:** ^1^ University of Edinburgh Centre for Inflammation Research, Queen’s Medical Research Institute, 47 Little France Crescent, Edinburgh, EH16 4TJ, UK.; ^2^ Department of Bioengineering, Rice University, Houston, TX, USA.; ^3^ The Roslin Institute, University of Edinburgh, Edinburgh, EH25 9RG.; ^4^ Edinburgh Clinical Research Facility, University of Edinburgh, Western General Hospital, Edinburgh, EH4 2XU, UK.

## Abstract

An intense, nonresolving airway inflammatory response leads to destructive lung disease in cystic fibrosis (CF). Dysregulation of macrophage immune function may be a key facet governing the progression of CF lung disease, but the underlying mechanisms are not fully understood. We used 5′ end centered transcriptome sequencing to profile *P. aeruginosa* LPS-activated human CF macrophages, showing that CF and non-CF macrophages deploy substantially distinct transcriptional programs at baseline and following activation. This includes a significantly blunted type I IFN signaling response in activated patient cells relative to healthy controls that was reversible upon in vitro treatment with CFTR modulators in patient cells and by CRISPR-Cas9 gene editing to correct the F508del mutation in patient-derived iPSC macrophages. These findings illustrate a previously unidentified immune defect in human CF macrophages that is CFTR dependent and reversible with CFTR modulators, thus providing new avenues in the search for effective anti-inflammatory interventions in CF.

## Teaser

Macrophages in Cystic Fibrosis exhibit intrinsic, CFTR-dependent, dysregulated type I IFN signalling following activation.

## INTRODUCTION

Cystic fibrosis (CF) is a life-limiting monogenic disease caused by loss-of-function mutations in the CF transmembrane conductance regulator gene, *CFTR*. The CFTR protein is an ion channel that functions to regulate the transepithelial movement of anions predominantly in mucus-secreting tissues, thereby acting to orchestrate the balancing of salt and fluid levels in mucus secretions (*1*). Disease-causing mutations in this gene affect multiple organs in CF including the gut, pancreas, and the liver, but it is the impact of dysregulated CFTR function in the airways that proves particularly harmful to individuals with CF. Dehydration of the airway surface liquid (ASL) and thickening of mucus secretions prevents effective airway mucociliary clearance and provides fertile ground for harmful bacteria, leading to recurring bouts of infection throughout life (*2*). An intense, neutrophil-dominated inflammatory response ensues that fails to resolve over time, driving chronic disease and causing permanent structural damage to the airways. Over the past 20 years, it has emerged that dysregulation of macrophage immune function is key in orchestrating the development of chronic inflammation in CF and subsequent disease (*3*–*5*). Among other phenotypes, CF macrophages exhibit impaired recognition and phagocytosis of bacteria (*6*–*10*), are hyperinflammatory following activation (*11*–*15*), and display inefficient clearance of apoptotic cells by efferocytosis (*10*, *16*, *17*). Therefore, macrophages can be seen as suboptimal drivers of innate immune defense and resolution of inflammation in CF. Furthermore, observation of inflammation in CF infants before any detectable infection raises the possibility that the genesis of CF inflammation is more complex than previously thought (*18*, *19*). Given also that healthy myeloid cells express functional CFTR (*20*–*22*) and with the replication of CF macrophage phenotypes in various total, and myeloid-specific, *Cftr* knockout (KO) animal models or through use of chemical CFTR inhibition in non-CF cells (*10*, *12*–*15*), we can hypothesize that in addition to secondary conditioning from the inflammatory milieu, macrophage dysfunction in CF is governed by intrinsic, CFTR-related factors. Investigating immune dysregulation in human CF macrophages and the underlying mechanisms can help inform novel treatments to combat harmful chronic inflammation in CF.

Much of the understanding surrounding macrophage behavior in CF was generated from animal models or with a focus on lung-resident alveolar macrophages and using a priori selected genes and proteins as readouts for assumptions about inflammatory phenotype. Recent single-cell RNA sequencing (RNA-seq) of CF patient sputum revealed that macrophage populations in the CF airways are predominantly monocyte-derived in origin, unlike embryonic-derived alveolar macrophages, which are dominant in healthy lungs (*23*). In this study, we use a high-resolution, unbiased approach to investigating human CF monocyte-derived macrophage (MDM) dysfunction. We deployed cap analysis of gene expression (CAGE) sequencing, a high-throughput method of promoter-level transcriptome profiling (*24*, *25*), to assess global transcriptional patterns in human CF MDMs following inflammatory activation. We identify novel dysregulation of immune signaling pathways in patient macrophages that are reversible upon correction of CFTR function in vitro with CFTR modulator triple therapy, Kaftrio/Trikafta, a triple therapy combining the potentiator ivacaftor (a compound that can rescue and enhance channel opening) with two additional CFTR correctors, elexacaftor and tezacaftor (pharmacological chaperones that aid protein folding and trafficking of the CFTR to the cell surface). The widespread transition of patients with CF to modulator therapies renders a study such as this, of “unmodulated” individuals, essentially unrepeatable. Hence, this dataset represents an important resource for the CF research community going forward. To further demonstrate the direct relation of the mutated *CFTR* genotype to the observed phenotype, we used CRISPR-Cas9 to correct the F508del mutation in patient-derived induced pluripotent stem cells (iPSCs), which were then differentiated to macrophages. Gene-edited (GE) iPS-macrophages show restored interferon (IFN) signaling, signifying the role of CFTR in said dysfunction and demonstrating the potential of gene editing methods to treat the dysregulated CF immune response.

## RESULTS

### Circulating monocytes exhibit heightened activation phenotype in CF

**Fig. 1.**
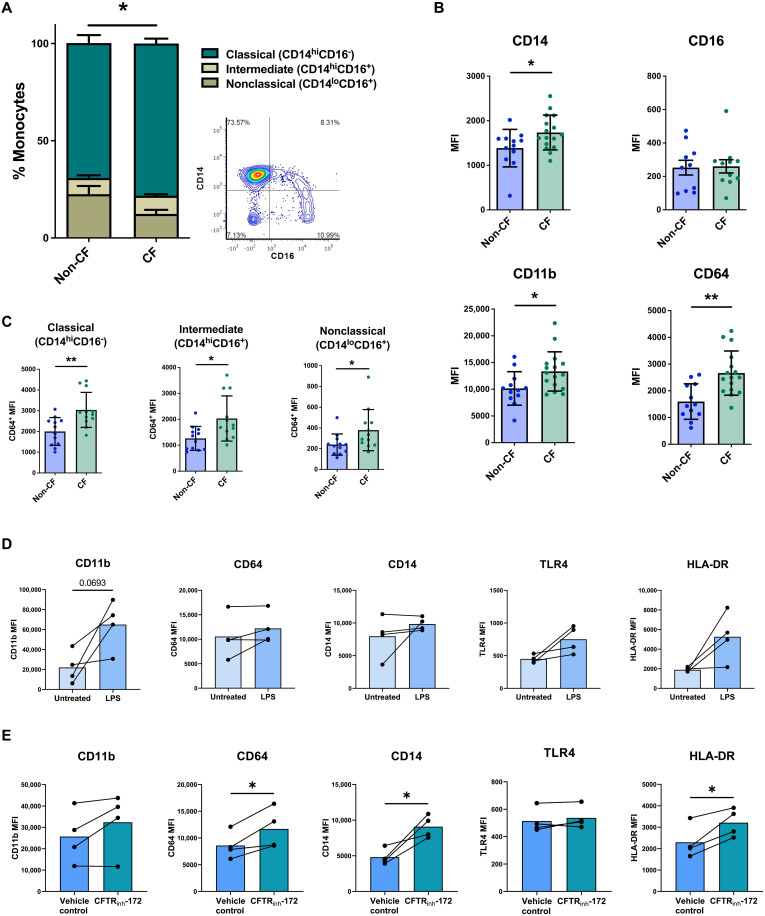
Heightened monocyte activation in CF circulation. (**A**) Comparison of monocyte subset proportions in CF and non-CF enriched monocyte populations (*n* = 13). Contour plot shows quadrant gating of the classical (CD14^+^CD16^−^), intermediate (CD14^+^CD16^+^), and nonclassical (CD14^−^CD16^+^) subsets. (**B**) Expression of activation markers on monocyte populations given as median fluorescence intensity (MFI). (**C**) CD64 MFI expression values on CF and non-CF monocytes, subdivided into the three subsets. (**D** and **E**) Whole blood from healthy donors was incubated at 37°C in the presence of *P. aeruginosa* LPS (100 ng/ml) or 10 μM CFTR_inh_-172 with untreated and vehicle [dimethyl sulfoxide (DMSO)] controls. After 4 hours, blood was stained directly for flow cytometry analysis of CD45^+^CD3^−^CD19^−^CD66b^−^CD56^−^CD14^+^HLA-DR^+^ monocyte populations for activation markers, given as MFI. *P ≤ 0.05 and **P ≤ 0.01. Obtained via unpaired and paired *t* tests, respectively. Data are presented as means ± SD.

Inflammation requires prompt recruitment of myeloid cells to the site of injury or insult with circulating monocytes infiltrating the tissue and assimilating into the resident macrophage pool (*26*). To track the onset of dysregulation in the CF macrophage immune phenotype and to query the relevant contribution of the diseased environment of the CF lung on such a phenotype, we first isolated monocytes from patient peripheral blood and compared the expression of known surface activation markers with non-CF controls. A significant difference was observed in the makeup of circulating monocyte populations in CF with a higher proportion of CD14^hi^CD16^−^ so-called classical monocytes and lower proportion of “nonclassical” (CD14^lo^CD16^+^) cells in total monocyte populations isolated from patient blood relative to non-CF controls (Fig. 1A). We also found that surface expression of CD14, as well as the CD64 Fc receptor and CD11b integrin, was significantly elevated on CF circulating monocytes compared to non-CF controls. This was not simply a result of there being a higher proportion of classical monocytes present in the population, as shown by CD64 expression, which was elevated in all three subsets from the patient samples, albeit to a lower extent in the intermediate and nonclassical populations (Fig. 1, B and C). Such augmented expression of the selected surface markers would indicate that monocytes exist in a higher state of activation in CF blood compared to non-CF (*27*–*30*). Given that intrinsic CFTR dysregulation is now known to affect effector function directly in certain immune cells, including monocytes, and considering also the inflammatory mediators present in human CF circulation, which includes heightened levels of circulating lipopolysaccharide (LPS), C-reactive protein (CRP), and calprotectin (s100A8/s100A9) (*31*–*33*), we questioned the extent to which the observed hyperactivation of circulating monocytes is due to intrinsic or extrinsic factors in CF. Healthy human blood was treated for 4 hours with LPS or the allosteric CFTR inhibitor, CFTRinh-172, and compared with untreated controls by whole-blood flow cytometry. LPS treatment induced demonstrable but not statistically significant increases in surface expression of CD11b, Toll-like receptor 4(TLR4), and human leukocyte antigen DR (HLA-DR; Fig. 1D), whereas CFTR inhibition prompted significant increases in HLA-DR, CD64, and CD14 expression relative to vehicle controls (Fig. 1E). While LPS is only a proxy for what is a vast and complex environmental inflammatory landscape in CF circulation, these data demonstrate that both exposure to microbial by-products of central CF disease and intrinsic loss of CFTR function can mimic the activated CF monocyte phenotype in healthy cells. What is not yet clear is the extent to which this affects the behavior of differentiated macrophage effector cells in the inflamed CF tissue.

### CAGE profiling of CF MDMs at baseline

Myeloid cell immune dysfunction in CF is not yet fully understood. In this work, we used an unbiased, high-resolution, transcriptomics profiling approach to probe the global transcriptional response of *Pseudomonas aeruginosa* LPS-activated CF MDMs and how this differs to non-CF controls. CAGE is a high-throughput method of promoter-level transcriptome profiling. The use of 5′ end–centered transcriptional profiling allows for genome-wide expression profiling and the capture of new layers of information regarding promoter usage, enhancer activity, and accurate identification of transcription factor binding motifs located in and around the active transcriptional start site (TSS) (*34*, *35*). In contrast to global gene expression analysis based on the full transcriptome sequencing following random fragmentation of RNA, CAGE involves sequencing of only a short segment of the 5′ end of each transcript following a process known as cap-trapping (fig. S1A) (*36*). This allows for an accurate measure of overall gene expression by collating TSS expression within each gene (fig. S1B) and for an in-depth assessment of the regulatory elements that drive transcription through analysis of the surrounding promoter region to a single-nucleotide resolution. Monocytes from healthy controls and from CFTR modulator-naïve, clinically stable patients with CF having at least one copy of the F508del CFTR mutation (individuals were either homozygous for F508del or heterozygous with a class I stop mutation; table S1) were isolated and cultured for 7 days in the presence of macrophage colony-stimulating factor (M-CSF; CSF1) to generate MDMs. CAGE library preparation and sequencing was then carried out on MDMs from both groups following 24 hours stimulation with *Pseudomonas* LPS (100 ng/ml) and untreated controls (fig. S1C).

Untreated and LPS-activated healthy control groups were first investigated to explore the transcriptional response of MDMs to LPS derived from the most clinically important bacterial pathogen affecting people with CF, *P. aeruginosa*, and to provide initial validation of the method itself by using it to examine a well-characterized immune interaction (fig. S2). This revealed 2042 genes to be significantly up-regulated in LPS-treated MDMs compared to untreated (LPSUP) and a similar number (2075) with increased expression in the untreated group relative to the treated samples (LPSDOWN; fig. S2D and data S2). Functional enrichment analysis of the top 398 genes [log fold change (logFC) ≥ 2; adjusted *P* value (*P*_ADJ_) ≤ 0.01] up-regulated by healthy MDMs in response to LPS (LPS_UP_) using KEGG (Kyoto Encyclopedia of Genes and Genomes) pathway analysis found the four most significantly overrepresented pathways in this dataset to be cytokine-cytokine receptor interaction (hsa04060; *P*_ADJ_ = 2.125 × 10–9), nuclear factor κB (NF-κB) signaling pathway (hsa06064; *P*_ADJ_ = 1.116 × 10–8), interleukin-17 (IL-17) signaling pathway (hsa04657; *P*_ADJ_ = 2.511 × 10–8), and tumor necrosis factor (TNF) signaling pathway (hsa04668; *P*_ADJ_ = 5.539 × 10^−8^; fig. S2E). This revealed a clear commonality between the processes most engaged by these cells as shown by CAGE and what would be expected on the basis of the well-characterized LPS-inducible MDM response (*37*, *38*).

**Fig. 2.**
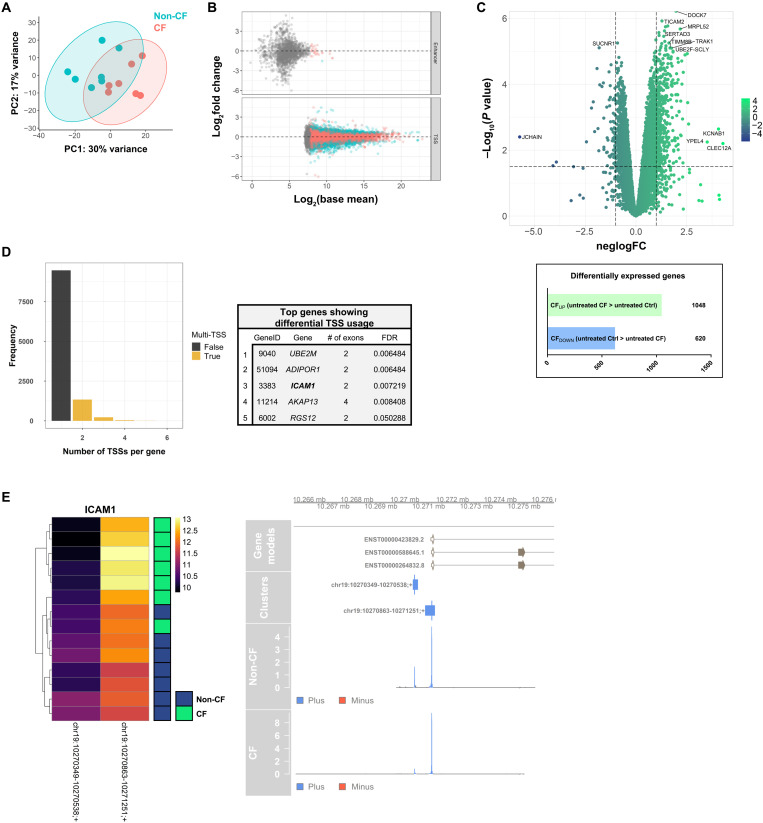
CAGE profiling of CF macrophages at baseline. (**A**) PCA based on CTSS expression across all samples (each dot represents a single sample; % variance shown on each axis title). (**B**) MA plot of differential uni- and bidirectional cluster expression between untreated non-CF and untreated CF macrophages. Blue points indicate a significantly differentially expressed gene with *P*_ADJ_ < 0.05. Gray points indicate a base expression level too low to enable differentially expressed analysis. (**C**) Volcano plot displaying all detected genes (11,073) following aggregation and Entrez Gene ID annotation of unidirectional tag clusters, by logFC and −log_10_*P* value. Dashed *y* intercept is at −log_10_*P* value of 1.5 (genes above this were considered statistically significant) with *x* intercepts at 1 and −1 logFC. The most highly significant and differentially expressed genes (logFC ≥ 1.5 and −log_10_*P* value ≥ 5, logFC ≥ 3.5 and −log_10_*P* value ≥ 2, logFC ≤ −3.5 and −log_10_*P* value ≥ 2, or logFC ≤ −1 and −log_10_*P* value ≥ 5) are labeled. Number of differentially expressed genes (*P*_ADJ_ <0.05) between groups listed below the plot. (**D**) The number of TSSs detected in each gene and the top 10 genes [by false discovery rate (FDR)] showing significant differential promoter usage between untreated CF and non-CF groups. (**E**) Heatmap displaying expression between untreated patient and control samples at active TSSs within the intercellular adhesion molecule–1 (ICAM1) gene and genome-browser visualization of promoter switching within the ICAM1 gene between treated and untreated groups.

Principal components analysis (PCA) based on overall TSS expression data revealed distinct separation on the PC1 axis (accounting for 30% variance) between non-CF and CF groups at baseline (Fig. 2A). Differential expression analysis at a TSS level revealed significant variance in expression of 2620 putative promoters, yet all but a few enhancers were expressed below the threshold, which allows for accurate detection of differential expression, with only 1 of the 28 enhancers above this threshold proving significantly different between the groups (Fig. 2B). TSSs expressed at ≥1 TPM (tags per million; normalized expression value) in more than two samples were annotated with gene models and totaled within each locus to produce a gene-level expression matrix. Even before activation, there were 1668 genes significantly differentially expressed (*P*_ADJ_ ≤ 0.05) between CF and control MDMs at baseline (Fig. 2C). Functional enrichment profiling using KEGG and Gene Ontology (GO) analysis of the most significantly differentially expressed genes (logFC ≥ 1; −log10*P* value ≥ 1) found no significant enrichment of terms related to biological processes. Such analyses lack sensitivity for identification of small networks of related gene products. However, examining the most significantly differentially expressed genes between the group showed that the top 10 genes more highly expressed in CF macrophages relative to non-CF (CF_UP_) included genes encoding proteins with key immune regulatory roles in macrophages. These included *TICAM2* (logFC = 1.57, *P* = 1.7 × 10^−6^) encoding TIR-domain-containing adaptor-inducing interferon-β (TRIF)–related adaptor molecule (TRAM), a key TLR signaling molecule, which also has a role in mediating macrophage phagocytosis (*39*, *40*), *NFKBIA* (logFC = 1.40, *P* = 2.4 × 10^−6^), and *CEBPB* (logFC = 1.13, *P* = 2.7 × 10^−6^; data S3) (*41*, *42*). The gene most significantly highly expressed in untreated non-CF cells relative to patient cells (CF_DOWN_) was *SUCNR1* (logFC = 1.81, *P* = 7.8 × 10^−6^), which encodes for the succinate receptor 1 (SUCNR1). Recent evidence has outlined an important role for SUCNR1 expressed on macrophages in regulating inflammatory activation and driving polarization toward an “M2-like,” pro-resolution phenotype following sensing of extracellular succinate, a known by-product of glycolytic metabolic rewiring of macrophages following inflammatory activation (*43*–*46*).

The presence of multiple TSSs in single genes provides cells with additional regulatory control over gene expression in different cell and tissue-dependent contexts and further expands the diversity of the human transcriptome through alternative promoter usage. Transcription from alternative promoters affects posttranscriptional modification and can result in various functionally distinct proteins produced from a single gene locus. Aberrant usage of certain alternative promoters has been associated with multiple cancers and several genes associated with Alzheimer’s disease, including the microtubule-associated protein tau gene (*MAPT*) (*47*, *48*). Analysis of promoter switching between untreated CF and non-CF MDMs found the presence of more than 1000 genes with two or more active TSS regions and four genes that exhibited significantly differential TSS usage between the two groups (Fig. 2D). A level of *ICAM1* (which encodes for an adhesion molecule implicated in macrophage polarization and efferocytosis) (*49*, *50*) transcription was shown to be initiated from a novel promoter-proximal TSS in non-CF MDMs. In contrast, CF cells used a downstream promoter located in the first annotated promoter of the gene as primary starting point of *ICAM1* expression (Fig. 2E). Substantially distinct gene expression programs active between CF and non-CF macrophages even after patient cells had been exposed to precisely the same culture conditions as healthy controls for 9 days indicate a deeply ingrained disease phenotype in patient cells, which, given the monogenetic nature of CF, suggests that it is linked directly to intrinsic loss of CFTR activity in macrophages.

### CAGE profiling of CF MDMs following *Pseudomonas* LPS activation

**Fig. 3.**
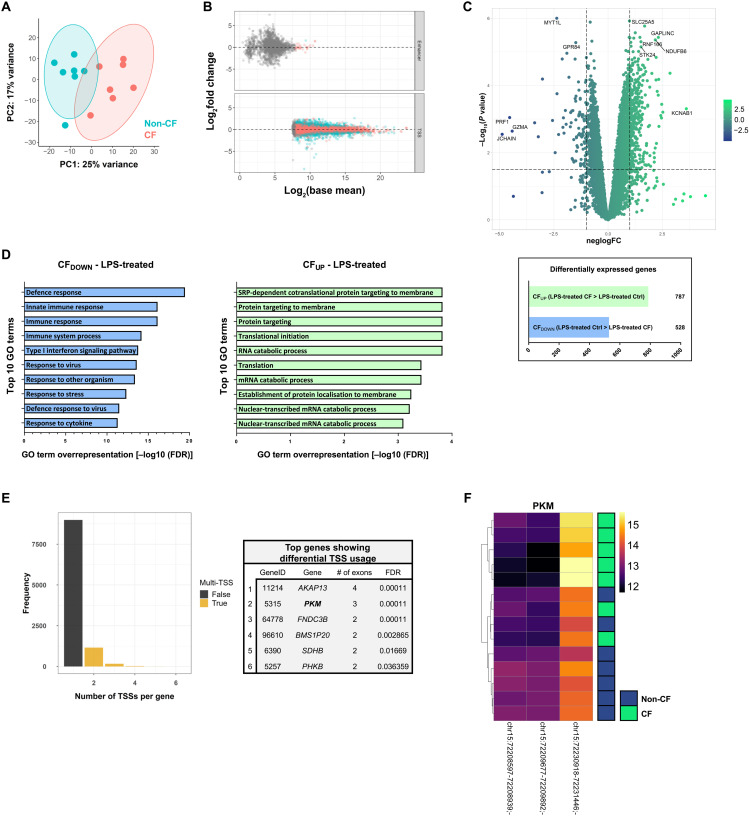
CAGE profiling of *P. aeruginosa* LPS-activated CF macrophages. (**A**) PCA based on global CTSS expression (each dot represents a single sample; % variance shown on each axis title). (**B**) MA plot of differential uni- and bidirectional cluster expression between 24-hour LPS-treated non-CF and CF macrophages. Blue points indicate a significantly differentially expressed gene with *P*_ADJ_ < 0.05. Gray points indicate a base expression level too low to enable differentially expressed analysis. (**C**) Volcano plot displaying all detected genes (10,342) following aggregation and Entrez Gene ID annotation of unidirectional tag clusters, by logFC and −log_10_*P* value. Dashed *y* intercept is at −log_10_*P* value of 1.5 (genes above this were considered statistically significant) with *x* intercepts at 1 and −1 logFC. The most highly significant and differentially expressed genes (logFC ≥ 1.5 and −log_10_*P* value ≥ 5, logFC ≥ 3.5 and −log_10_*P* value ≥ 2, logFC ≤ −3.5 and −log_10_*P* value ≥ 2, or logFC ≤ −1 and −log_10_*P* value ≥5) are labeled. Number of differentially expressed genes (*P*_ADJ_ <0.05) between groups listed below the plot. (**D**) The top 10 significantly enriched GO biological processes (measured by FDR) from the top 339 genes more highly expressed in LPS-treated CF samples relative to LPS-treated non-CF (CFUP); criteria for “top” genes was logFC > 1 and −log_10_*P* value > 1. (**E**) The number of TSSs detected in each gene and the top 10 genes (by FDR) showing significant differential promoter usage between LPS-treated CF and non-CF groups. (**F**) Heatmap displaying expression between untreated patient and control samples at active TSSs within the PKM gene.

CAGE libraries were then compared between CF and non-CF MDMs following 24 hours of stimulation with *P. aeruginosa* LPS (100 ng/ml), which served as a basic in vitro proxy for the kind of inflammatory/microbial challenge that may be encountered by differentiating macrophages upon entry to the inflamed CF airways. Clear variance was seen again between patient and control TSS expression by PCA (Fig. 3A). Analysis of TSS expression revealed differential expression between activated CF and non-CF groups at 2115 sites, but most enhancers were discarded before the final analysis because of low detection (Fig. 3B). Following gene-level clustering of tag clusters, differential gene expression analysis revealed 787 genes with significantly higher expression in activated CF samples relative to activated healthy controls (CFUP) and 628 genes with significantly lower expression in activated CF samples relative to activated healthy controls (CFDOWN) (Fig. 3C and data S4). Of the 1315 total genes found to be differentially expressed between LPS-stimulated CF and healthy control samples, 681 genes (51.8% of all significant differences) were also significantly differentially expressed between CF and non-CF samples in the untreated, baseline comparison. Enrichment analysis of GO biological processes related to the top 339 CFUP genes (logFC ≥ 1; −log10*P* value ≥ 1) showed enrichment of various metabolic processes including RNA catabolic processes, as well as protein targeting and signal recognition particle (SRP)–dependent protein targeting (Fig. 3D). SRP-dependent signaling of misfolded proteins in the ribosome initiates the unfolded protein response (UPR), which leads to degradation of mutant mRNAs and clearance of misfolded proteins within the cell (*51*). All CF samples were derived from patients with at least one class II CFTR mutation resulting in amino acid deletions and missense mutations that lead to the production of an abundance of misfolded mutant CFTR protein, so UPR would therefore be expected. More than 1000 genes active in the LPS-treated samples contained at least two active TSSs, with six genes demonstrating significant promoter switching between activated CF and non-CF groups (Fig. 3E). The *PKM* gene had three active TSSs with CF cells showing preferential use of the first (reading 5′ to 3′) of the three, relative to non-CF controls (Fig. 3F). There are at least 12 known functionally separate isoforms of pyruvate kinase (PKM), reported as being produced by alternative splicing, with switching between the major isoforms from M1 (PKM1) to M2 (PKM2) implicated in tumor cell growth, with the latter being a central driver of aerobic glycolysis in tumors and other highly proliferating cells (*52*).

**Fig. 4.**
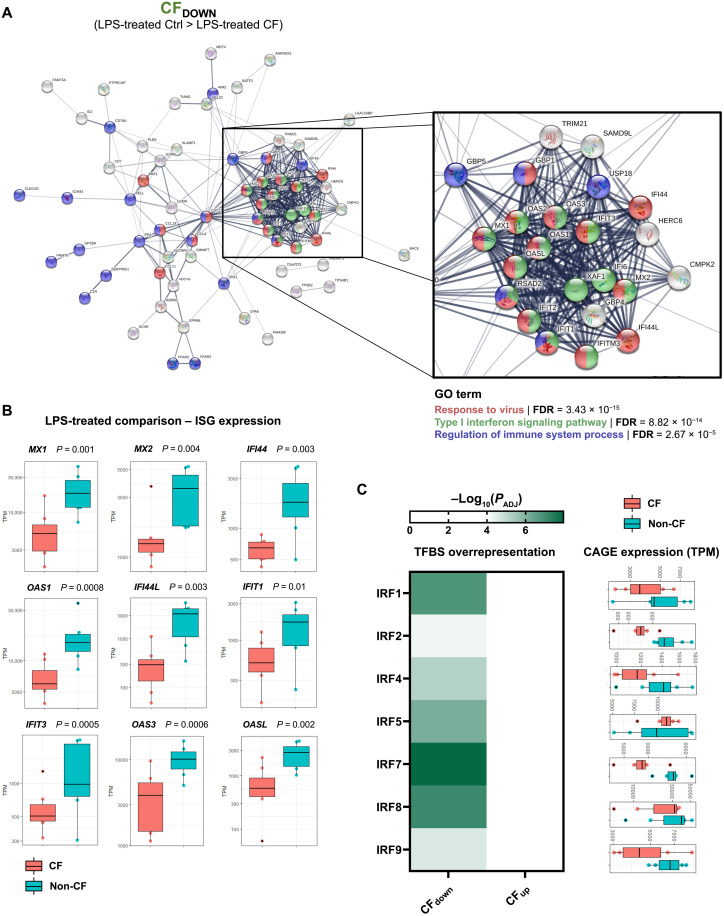
Blunted type I IFN signaling in activated CF macrophages. (**A**) STRING network analysis of the top genes in the CF_DOWN_ set (100). Gene products related to selected GO biological processes are colored as shown. (**B**) Individual plots of differential TSS expression between activated patient and control within selected ISGs. *P* values are shown. (**C**) Enrichment of TFBSs for IRFs within 500 bp of the primary TSS regions of the top 100 LPS-treated CF_DOWN_ genes. Also shown is the individual TSS expression of each IRF and the respective motif sequences, complementary of the IFN-stimulated response element (ISRE) TFBS consensus region: AGTTTCNNTTTCN.

GO functional enrichment analysis of the top CF_DOWN_ genes (logFC > 1 and −log_10_*P* value > 1; 100 genes) revealed significant enrichment of various biological processes related to the innate immune response (Fig. 3D). Search Tool for Retrieval of Interacting Genes/Proteins (STRING) network analysis helped visualize functional interactions of gene products and showed a distinct cluster of genes with numerous close interactions, suggesting dysregulation of an entire process or pathway (Fig. 4A). Most of the genes in this cluster related to the cellular response to virus and the type I IFN signaling pathway. Analysis of predominant TSS expression in several major IFN-stimulated genes (ISGs), the products of type I IFN signaling, showed significantly lower expression of ISGs in LPS-activated CF MDMs relative to activated non-CF controls (Fig. 4B). None of the ISGs shown were differentially expressed at baseline between the untreated patient and healthy control macrophages, suggesting that this disparity is an effect of LPS activation (data S3). The knowledge of TSS expression to a single nucleotide resolution was used to accurately determine enrichment of DNA binding motifs around active promoters and predict transcription factor binding activity. Sequences 500 base pairs (bp) immediately upstream and downstream of the top 100 CFDOWN TSSs were extracted and analyzed for the presence of transcription factor binding sites (TFBSs) and enrichment of such sites across the 100 genes. This revealed significant overrepresentation of several IFN regulatory factors (IRFs) that regulate transcription of IFNs and ISGs, immediately surrounding the most significant LPS-treated CFDOWN TSSs (Fig. 4C). TSSs located adjacent to IRF7 binding sites were particularly enriched in the top CFDOWN TSSs, and expression of the primary *IRF7* promoter itself was also significantly reduced in activated CF MDMs relative to non-CF controls (chr11:615877-616084; logFC = 1.33, *P*_ADJ_ = 0.00032; data S4). Along with IRF3, IRF7 is the key IRF responsible for regulating transcription of type I IFNs following activation of MyD88-independent, TRIF-dependent signaling pathways that are triggered by various receptor-ligand binding interactions, including LPS-induced TLR4 signaling (*53*).

### In vitro correction of CFTR function augments IFN signaling in activated CF macrophages

CFTR modulators act by chemically correcting CFTR activity in CF cells and the most recent therapy approved for clinical use, Kaftrio (aka Trikafta; elexacaftor-tezacaftor-ivacaftor) was shown in phase 3 clinical trials to induce a 14.3% increase in predicted lung function (FEV1) and a 63% reduction in the rate of pulmonary exacerbation at 24 weeks, in patients heterozygous for F508del and a minimal-function mutation relative to the placebo control group (*54*). Kaftrio is highly effective in treating not only individuals with two copies of the F508del mutation but also those with one copy and an additional class I, minimal function mutation (e.g., G542X), accounting for a large number of individuals with the most severe disease and meaning that modulators are now in place for up to 95% of the CF population in the United Kingdom (*55*).

**Fig. 5.**
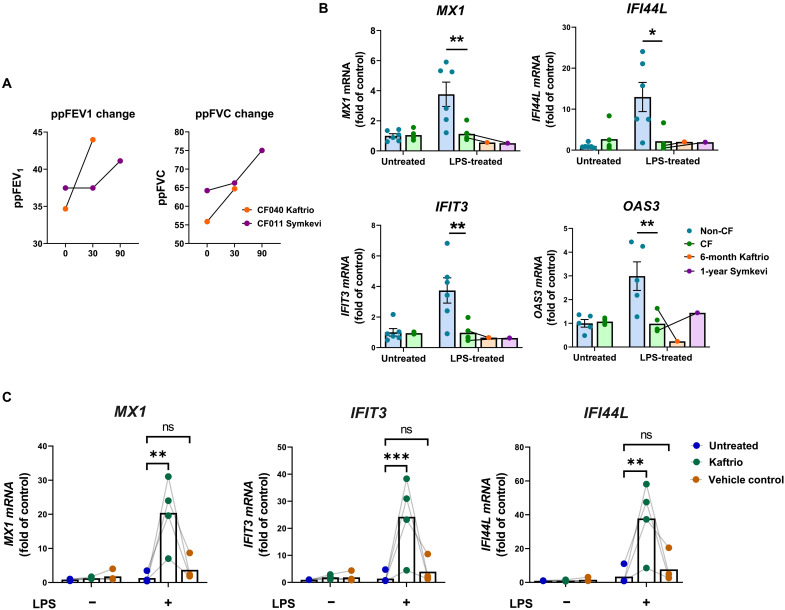
Dysregulated type I IFN signaling in activated CF macrophages is rescued with CFTR correction using CFTR modulators. (**A**) Changes to ppFEV_1_ (percent predicted forced expiratory volume) and ppFVC (percent predicted forced vital capacity) of two patients included in CF sample group for CAGE sequencing after 30 and/or 90 days of modulator use. (**B**) ISG expression following 24-hour treatment with *P. aeruginosa* LPS (100 ng/ml) displaying pretreatment samples with paired (shown by lines), follow-up samples from modulator-treated individuals. Analyzed by SYBR Green qRT-PCR (quantitative reverse transcription polymerase chain reaction) at 24 hours after treatment. Expression values were calculated relative to 18*S* expression using the 2^–ΔΔ*C*t^ method and given as fold of untreated non-CF controls. *P ≤ 0.05 and **P ≤ 0.01. Obtained via two-way analysis of variance (ANOVA) with Sidak’s multiple comparisons test. Data are presented as means ± SD. (**C**) Expression of key ISGs by CF MDMs following 24 hours of LPS stimulation after being differentiated in vitro with or without supplementation with CFTR modulator, Kaftrio, or vehicle control (0.11% DMSO). ns, not significant.

Repeat blood samples were acquired from two patients whose cells had been used in the initial CAGE experiment but had subsequently started long-term use of CFTR modulator therapies, enabling comparison of pre- versus post-modulator treatment MDMs. Lung function data revealed that both individuals responded well to their respective treatments with improvements in ppFEV1 and ppFVC over the first 90 days (Fig. 5A). Although the patients were receiving CFTR modulator therapy when the second blood samples were collected, their MDMs were initially cultured ex vivo in the absence of these modulators (as the modulators were not available for experimental use at that time). The same LPS treatment as before was carried out to examine the impact of reduced disease severity and patient modulator therapy on the blunted expression of ISGs in activated patient MDMs. ISG expression levels in MDMs from patients receiving either Kaftrio (ivacaftor/tezacaftor/elexacaftor) for 6 months or earlier generation therapy Symkevi (ivacaftor/tezacaftor) for 12 months were not altered relative to paired baseline levels and remained significantly reduced compared to activated non-CF samples (Fig. 5B). Any effects of modulators on the isolated monocytes may have worn off during MDM differentiation in culture; therefore, this was not necessarily reflective of the contribution of CFTR dysfunction to the initial observation. It was, nonetheless, suggestive that either the inflammatory nature of CF circulation was not altered with modulator use or it was altered but offered little secondary effect on monocyte/macrophage immune function.

Subsequent acquisition of Kaftrio (provided by Vertex Pharmaceuticals) for experimental use next enabled assessment of the contribution of CFTR dysfunction on blunted immune signaling in activated CF macrophages by culturing patient cells in the presence or absence of CFTR modulators in vitro. Given the widespread rollout of these drugs in the United Kingdom by this time, it was only possible to obtain blood samples from patients already enrolled on daily Kaftrio therapy, with no significant number of “noncorrected” individuals available. Monocytes were therefore obtained from modulator-treated individuals and cultured either with the drug supplemented in vitro or in “wash-out” conditions in the absence of correction. Given the short half-life of modulators requiring twice daily use for patients, it was reasoned that the effects of Kaftrio on patient cells would be lost during the 9-day culture (compatible with the data in Fig 5B) and therefore allow for a comparison between corrected and noncorrected macrophages. CF MDMs differentiated in the presence of Kaftrio showed significantly increased expression of key ISGs—*MXI*, *IFIT3*, and *IFI44L*—following 24-hour *P. aeruginosa* LPS activation, relative to noncorrected, wash-out cells and relevant vehicle control [0.11% dimethyl sulfoxide (DMSO); Fig. 5C]. These data implicate intrinsic CFTR dysfunction in causing the blunted type I IFN signaling response seen in LPS-activated CF MDMs and show that clinically relevant CFTR modulators have the potential to rescue this phenotype in patient cells.

### Gene editing the F508del mutation in patient-derived iPS-macrophages corrects the IFN signaling CF phenotype

**Fig. 6.**
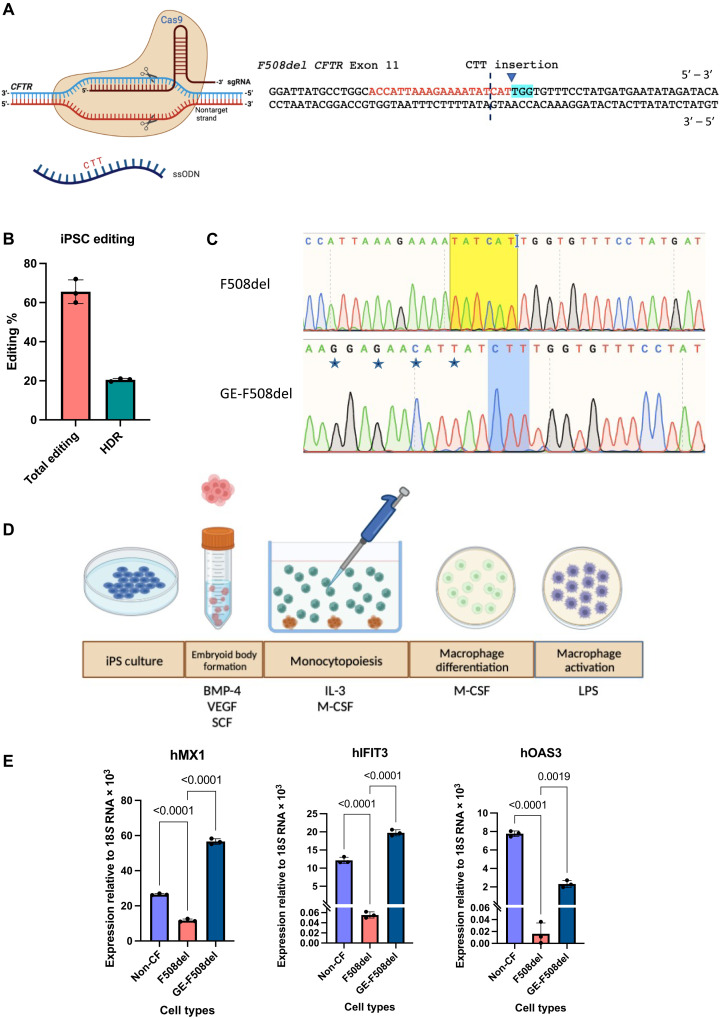
Gene editing restores the IFN signaling in iPS-derived macrophages. (**A**) Schematic of CRISPR-Cas9 with an ssODN donor template containing CTT to correct the F508del mutation by targeted insertion of the ssODN donor at the Cas9-induced DNA double-stranded break. The CTT insertion site is marked with a triangle in the exon 11 sequence with the single gRNA (sgRNA) target sequence in red and corresponding PAM site highlighted in blue. The CTT is centered on the donor template, and the ssODN donor is complementary to the target strand of the DNA. (**B**) CRISPR-Cas9–based gene editing achieved 20% allelic correction of the F508del mutation in CFTR using the designed sgRNA and ssODN donor. (**C**) The top chromatogram shows the sequencing results of the patient-derived iPSCs where the CTT will be inserted after the yellow highlighted region. The bottom chromatogram shows the GE-F508del cell sequence with the CTT insertion; after gene editing, the cells were sorted to expand a clone with homozygous repair. We also introduced four silent mutations, as starred on the chromatogram, to prevent recutting of the F508del locus after repair. (**D**) The three cell types (non-CF, F508del, and GE-F508del) were then differentiated to macrophages using the procedure shown here where the iPSCs were first grown in AggreWell 800 plates to form embryoid bodies (EBs) under the influence of bone morphogenetic protein 4 (BMP-4), vascular endothelial growth factor (VEGF), and SCF before plating it on the wells for monocytopoiesis induced by IL-3 and M-CSF. The monocytes were harvested from the media and plated for differentiation to macrophages using M-CSF. The terminally differentiated cells were stimulated for 24 hours with LPS. (**E**) Macrophages were harvested for qRT-PCR analysis of ISG expression (two-way ANOVA: Dunnett’s multiple comparisons test). Schematics in (A) and (D) were created on Biorender.com.

The plasticity of the macrophage’s response to environmental cues makes it difficult to delineate effects of intrinsic genetic defect from the influence of the environment on the response of the CF macrophages. To overcome this challenge and enable study of CF macrophage behavior independently from the influence of the environment, we used iPS-differentiated macrophages from (i) patient-derived CF iPSCs, (ii) non-CF individual-derived iPSCs, and (iii) GE iPSCs. Using the powerfully precise technology of CRISPR-Cas9, we corrected the F508del mutation in patient-derived CF iPSCs (Fig. 6A). By using the protospacer adjacent motif (PAM) site proximal to the CTT deletion, we used single-stranded oligodeoxynucleotide (ssODN) to insert the CTT with allelic homology-directed repair (HDR) rate of 20% (Fig. 6B). To prevent recutting of the same locus, we also induced silent mutations close to the cut site such that mismatch between guide RNA (gRNA) and DNA sequence after repair will prevent the gRNA recutting at the corrected site. After the editing, cells were singly sorted and genotyped to isolate clones with homozygous repair of the F508del locus (Fig. 6C).

The three types of iPSCs (non-CF, F508del, and GE) were then differentiated using well-established protocols (*56*) to derive macrophages (Fig. 6D). Non-CF and F508del iPS-macrophages, when stimulated for 24 hours with LPS, showed significant differences in expression of several ISGs that were differentially expressed between patient and control activated MDMs (Fig. 4B and fig. S3). The LPS-activated GE iPS-macrophages exhibited IFN signaling closely resembling that of the non-CF iPS-macrophages, where ISG expression was similar to control cells (Fig. 6E).

## DISCUSSION

In this study, we demonstrate a novel immune defect in LPS-activated human CF macrophages and show that this disease phenotype is reversible by the actions of CFTR modulator therapies, thus also underlying the role of intrinsic CFTR dysfunction in causing dysregulation of immune function in CF myeloid cells. We also showed that this immune defect was present in macrophages from CF patient-derived iPSCs. By correcting the most common CF-causing gene mutation F508del using CRISPR-Cas9 gene editing, we observed a reversal of this immune defect in LPS-activated macrophages differentiated from GE CF iPSCs. Macrophage immune dysfunction in CF is well established (*3*, *4*, *57*), but the extent of this dysfunction remains unclear, as well as the fundamental mechanisms that produce this phenotype and what the relative contribution is of secondary acquired factors (exposure of the cells to systemic inflammation and a chronically inflamed/infected lung) and primary, intrinsic (loss of functional CFTR in myeloid cells) causal factors. To address this, we acquired peripheral blood monocytes from clinically stable patients with CF (i.e., not from individuals who were exacerbating, receiving intravenous steroids or antibiotics, or who were posttransplant) and from healthy controls and generated MDMs in vitro with no autologous serum used, thus reducing the exposure of patient cells to said acquired disease factors, given that, at the time of acquisition, CF monocytes are unlikely to have been exposed to the inflammatory milieu of the diseased lung.

Analysis of surface marker expression on monocytes by flow cytometry revealed heightened expression of CD64, CD11b, and CD14 on CF monocytes relative to non-CF. Increased expression of such markers is a well-established indication of elevated activation/maturation, with CD11b for example, an integrin that is necessary for optimal locomotion of monocytes along the luminal surface of the endothelium to sites where extravasation can occur (*27*–*30*). Chemical inhibition of CFTR activity in healthy human monocytes using an allosteric CFTR inhibitor (CFTR_inh_-172) induced increased surface expression of CD64 and CD14 while LPS, elevated in patient serum (*31*, *58*) and therefore used as an in vitro proxy for the inflamed CF circulation, caused a trend toward heightened CD11b expression. It is important to exercise caution when interpreting CFTR_inh_-172–induced differences as entirely CFTR dependent given the multiple known off-target effects of this inhibitor, including increased induction of NF-κB signaling independently of CFTR inhibition (*59*, *60*). A more targeted approach, including using CRISPR-Cas9 gene editing, is far preferable. Also shown was a difference in the subset composition of patient circulating monocyte populations, which is a higher proportion of CD14^hi^CD16^−^ “classical” monocytes seen in CF blood relative to healthy controls. In humans, classical monocytes circulate for around a day before either extravasating and trafficking to the tissue or, alternatively, undergoing conversion to longer-lived nonclassical cells (*26*). One explanation for the differences in subset percentages in CF may be that constant inflammatory stimuli, primarily sourced from the diseased airways, may necessitate more frequent trafficking of classical monocytes to the tissue, therefore blocking the normal transition of classical to nonclassical cells that occurs during homeostasis. Together, these data suggest that monocytes are hyperactivated in CF circulation even before exposure to the diseased tissue due to conditioning by a combination of intrinsic and environmental factors. We next questioned what effect this has on the function of macrophages that arise from these cells in the CF airways.

CF and non-CF MDMs were activated for 24 hours in the presence of *P. aeruginosa* LPS, before RNA extraction and transcriptome profiling by 5′ end–centered CAGE sequencing. *P. aeruginosa* LPS was chosen as a stimulant given the prominence and clinical importance of this pathogen in individuals with CF, primarily from early adulthood at which point disease progression often accelerates. This served as a useful and replicable in vitro challenge that could mimic precisely the major inflammatory stimulant that greets infiltrating immune cells in the CF lung. However, it is important to consider the wider complexity of the diseased CF airways, being far more diverse than any one microbial product, as well as the innate plasticity of macrophages as limitations on our ability to draw comprehensive conclusions from this study as to the transcriptional response of macrophages in vivo. Even at baseline, there were substantial disparities in gene expression patterns of CF and non-CF macrophages with a total of 1668 differentially expressed genes between the two groups. The gene most highly expressed in untreated healthy controls relative to untreated CF cells (CF_DOWN_), *SUCNR1*, encodes for the SUCNR1. Succinate accumulates in inflammatory macrophages as a by-product of a broken Krebs cycle during the reprogramming of macrophage metabolism from oxidative phosphorylation to glycolysis and feeds into inflammatory pathways, enhancing the expression of *IL1*β (*45*). Inflammatory activation also causes release of succinate into the extracellular space where it initiates auto- and paracrine binding to SUCNR1 on local macrophages. Extracellular succinate can also function to favor the colonization and expansion of several microorganisms that favor catabolism of succinate as a preferred carbon source, including *P. aeruginosa* (*61*). Whereas intracellular binding of succinate initiates a proinflammatory signaling program via HIF-1α, there is now evidence that extracellular binding, via SUCNR1, serves instead as an “alarmin” and initiates a strong anti-inflammatory response with up-regulation of a distinctly M2-like macrophage transcriptional program (*43*, *44*). Hence, a myeloid-specific knockout of *Sucnr1* in mice led to local tissue inflammation and development of obesity (*43*). The 5′ end–centered nature of CAGE allowed for the investigation of differential promoter usage between groups and revealed multiple genes from which transcription was preferentially initiated at different sites in CF MDM at baseline compared to non-CF MDM. This included intercellular adhesion molecule–1 (ICAM1), the protein product of which, ICAM1 (aka CD56), is an adhesion molecule implicated in macrophage polarization and efferocytosis and known to have several functionally distinct isoforms, with preferential expression of some isoforms more than others reported as contributing to disease progression (*49*, *50*, *62*). Such studies refer to alternative splicing of known exons, but the results observed in this study suggest that the longer isoforms may originate instead by promoter switching from the novel TSS observed here, upstream of the first annotated promoter. ICAM1 clustering at the surface of inflammatory macrophages has recently been shown to mediate binding to, and uptake of, apoptotic cells, implying an important role for the molecule in macrophage-driven resolution and repair (*63*). It remains to be seen whether the differential TSS usage in CF *ICAM1* leads to functionally distinct protein isoforms and whether this has an impact on the ability of these cells to carry out efferocytosis, a process known to be inefficient in CF inflammation generally.

Similar transcriptional divergence between CF and non-CF MDM was also seen after 24 hours of LPS stimulation, with 1315 genes significantly differentially expressed between the two, 681 of which (51.8%) were also differentially expressed at baseline in the untreated comparison. Unbiased functional enrichment analysis using STRING revealed that ISGs, including *MX1*, *MX2*, *IFIT1*, *IFIT3*, *OAS1*, *OAS3*, *OASL*, *IFI44*, and *IFI44L*, made up a significant proportion of the top genes that were most highly expressed in activated healthy control MDMs relative to CF cells (CF_DOWN_). The well-established antiviral effector functions of IFNs and ISGs would suggest that a suboptimal type I IFN signaling response, as described, may have a detrimental impact on the immune defense against viral pathogen. While people with CF are typically no more susceptible to viral infections than the general population, there is evidence that acute respiratory viral infections are particularly harmful to patients with CF, having data showing that the majority of hospital admissions for pulmonary exacerbations of adult patients with CF are preceded by viral infections, albeit from a low sample size (*64*). The antiviral immune response has been overlooked in CF research, likely because of the prominence of bacterial infections from a clinical perspective, but a prospective study of CF infants found high prevalence of viral respiratory tract infections (particularly rhinoviruses, influenza virus, and coronaviruses) and an association of such infections with bacterial inflammation, suggesting that the acquisition of viral respiratory infections, particularly early in life, may contribute to laying the groundwork for the initiation of bacterial inflammation in CF (*64*). Moreover, type I IFN signaling plays a central role in the macrophage response to bacterial infection, and it can produce protective or deleterious effects, dependent on the tissue- and infection-specific context (*65*). This includes the ability of macrophage-derived IFN-β to orchestrate the resolution of bacterial inflammation in the lung by reprogramming local macrophage populations toward a pro-resolving phenotype with enhanced efferocytosis of apoptotic neutrophils (*66*). Further work is required to determine the extent of dysregulated IFN signaling in CF (e.g., does it extend to other immune cells and epithelial cells as previous studies suggest) and to elucidate the full extent to which this phenotype affects the optimal immune response to viral and bacterial infection.

While dysregulated CFTR-dependent type I IFN signaling has not previously been shown in CF macrophages, there have been hints of intrinsic changes to this signaling pathway in other cell types in CF. MicroRNA-specific RNA-seq of human CF MDMs revealed significantly increased expression of miR-146a, the dysregulation of which is implicated in numerous inflammatory diseases, and GO term analysis of gene targets of this microRNA revealed significant enrichment of genes involved in type I IFN signaling and the cellular response to type I IFN (*67*). Dampened up-regulation of OAS1 protein in response to IFN-γ or poly I:C (double-stranded RNA synthetic analog: TLR3 agonist) has been shown in CF human airway epithelial cells, obtained from brushing of explanted lungs, relative to non-CF controls (*68*). Likewise, impaired activation of type I IFN signaling response has been demonstrated in a CF human bronchial epithelial cell line, IB3-1 (*69*). IB3s are heterozygous for F508del and W1282X (class I nonsense mutation) and were shown to exhibit a depleted type I IFN signaling response to infection with *P. aeruginosa* and in response to 2 hours of LPS activation, with diminished expression of various ISGs relative to the CFTR-corrected control cell line, C38. The presence of this phenotype in an epithelial cell line with human CF mutations suggests a clear role for CFTR in the induction of type I IFN signaling.

To determine the intrinsic versus acquired nature of dysregulated IFN signaling in CF MDMs, we carried out an experiment whereby patient MDMs were differentiated either in the presence or in the absence of CFTR-correcting Kaftrio, before LPS-activating the fully differentiated cells as before and assessing ISG expression by quantitative reverse transcription polymerase chain reaction (qRT-PCR). CF MDMs that were cultured in the presence of CFTR modulators showed significant reductions in ISG expression following LPS stimulation relative to activated MDMs from the same patients that were differentiated in the absence of CFTR correction. Further investigation of the importance of CFTR in the process of macrophage differentiation, with modulators provided both at the monocyte, intermediate, and MDM stage before LPS challenge would help with better understanding the role of CFTR in steady-state macrophage functioning. With the advent and ease of CRISPR-Cas9–based genome editing, we then used this technique to correct the F508del mutation in patient-derived iPSCs, thus illustrating the genotype dependence of the ISG expression in iPS-derived macrophages. The non-CF and CF cells showed significant differences in IFN signaling, which was then resolved after repairing the F508del mutation. The data shown in this study are consistent with a direct role for macrophage CFTR deficiency in causing perturbed type I IFN signaling in the same cells. The use of CRISPR-Cas9 editing to knockout CFTR in healthy human MDMs has been demonstrated previously and was shown to induce development of multiple known CF macrophage functional defects (including reduced bactericidal ability), strongly suggesting the CFTR-dependent nature of these impairments (*70*). It is perhaps worth noting that the KO was carried out after differentiation to MDMs and could not therefore mimic the effects of loss of CFTR function in precursors and differentiating cells. The restorative capabilities of CFTR modulators on CF macrophages directly in vivo, or the effects that such treatments are having on known CF macrophage phenotypes such as those highlighted in this study are not yet fully known. Evidence of restoration of various CF-related functional impairments (defective bacterial killing and efferocytosis) in patient MDMs following Kaftrio treatment course was reported recently, although there were no such changes in inflammatory cytokine secretion in response to the treatment (*71*). Further work is required to determine the extent to which modulator-related changes to immune cell function occur in vivo, but the notion that such therapies can alter patient macrophage function and direct it toward a healthy phenotype is promising, particularly in the context of aiming to counteract harmful nonresolving CF inflammation, in which macrophages are heavily involved. This may also have potential for impact beyond the world of CF, given that it is now well established that acquired CFTR dysfunction is an important feature of smoking-related lung diseases, with evidence that cigarette smoke can impair bacterial phagocytosis in a CFTR-dependent manner in RAW264.7 cells, while CFTR modulators can improve the “CF-like” clinical feature (mucus thickness/stasis and bronchiectasis) of animal models of Chronic Obstructive Pulmonary Disease (COPD) (*72*–*75*). If it was that CFTR correctors are not sufficient to modulate monocytes and macrophages in vivo, then one potential future avenue for therapy to combat inflammation may well be the differentiation of patient monocytes in vitro, in the presence of modulators, before autologous transfer of corrected, MDMs back to patients with the aim of aiding a swift, pro-resolution, pro-repair immune cascade.

In this study, we have identified novel, intrinsic dysregulation of a crucial innate immune signaling pathway in activated human CF MDMs. Furthermore, we have shown that this phenotype is CFTR-dependent, amenable to, and reversible upon, treatment with the newest generation of CFTR modulator therapies in vitro. This underlines the potential for macrophage targeting in the search for an elusive, effective anti-inflammatory treatment strategy to combat chronic disease and promote lasting resolution and repair in CF.

## MATERIALS AND METHODS

### Study participants

Peripheral blood was collected from patients with CF attending the Scottish National CF Service adult clinic at the Western General Hospital, Edinburgh. Both patient and health donor demographics used for the CAGE experiment are detailed in table S1. Only patients considered to be in a clinically stable condition (no intravenous antibiotics required in the 2 weeks before sample collection and no signs of exacerbation) were included, while lung transplant recipients were excluded. All patients gave written consent, and this study was approved by the East of Scotland Research Ethics Committee (15/ES/0094). Peripheral blood was also collected from age- and sex-matched healthy adult volunteers, who provided written consent under the ethical agreement code, AMREC 15-HV-013, under the project number, CIRBRP009.

### Isolation of human peripheral blood mononuclear cells and monocyte enrichment

Peripheral blood mononuclear cells (PBMCs) were isolated using Percoll (GE Healthcare) density gradient centrifugation. Human peripheral blood was collected into 3.8% sodium citrate and centrifuged for 20 min at 350*g*. Following aspiration of plasma, leukocytes were separated from erythrocytes by 6% dextran sedimentation for 30 min and washed in 0.9% saline. PBMCs were then isolated by a discontinuous Percoll gradient of 81, 70, and 55% with the cells resuspended in 55% Percoll (Sigma-Aldrich) and carefully layered onto the 70% layer. The gradient was then centrifuged for 20 min at 700*g* (20°C, brake 0, acceleration 0), and PBMCs were collected using a Pastette from the 55/70 interface. These cells were then washed twice in cation-free 1× Dulbecco’s phosphate-buffered saline (DPBS; Thermo Fisher Scientific). Monocytes were isolated from the PBMCs by MACS cell separation using a pan-monocyte, negative selection magnetic beads according to the manufacturer’s instructions (Miltenyi Biotec, #130-096-537).

### Monocyte culture and treatment

For analysis of human circulating monocytes, following isolation from peripheral blood, 0.25 × 10^6^ monocytes were stained and fixed in 4% paraformaldehyde (PFA; Sigma-Aldrich) for profiling by flow cytometry. For ex vivo treatments of monocytes directly in whole blood, 100 μl of peripheral blood was added to a 5-ml polystyrene fluorescence-activated cell sorting (FACS) tube (Fisher Scientific). Blood was treated with either *P. aeruginosa* 10 LPS (100 ng/ml; Sigma-Aldrich), 10 μM CFTRinh-172 (Sigma-Aldrich), or 0.04% DMSO (Sigma-Aldrich) as vehicle control and incubated for 4 hours at 37°C. At this point, blood was stained directly for flow cytometry.

### Flow cytometry

For flow cytometry analysis of isolated monocyte populations, 0.25 × 10^6^ cells were harvested for phenotypic analysis. For analysis of monocyte populations in whole blood, staining was performed directly onto 100 μl following 4-hour treatment. Red cells were lysed from human whole blood using BD FACS Lysing Solution 10X (Fisher Scientific) and diluted 1:10 in nuclease-free water for 10 min. Samples were washed with PBS^−/−^ and stained for 25 min at 4°C, protected from light, with a panel of fluorochrome-labeled antibodies for monocyte phenotypic and functional surface markers: CD56 (HCD56), CD19 (H1B19), CD3 (HIT3a), CD66b (G10F5), CD64 (10.1), CD16 (3G8), CD11b (ICRF44), TNFR1 (W15099A), TNFR2 (3G7A02), CD14 (63D3), HLA-DR (L243), CD11c (3.9), TLR4 (HTA125), and CD45 (2D1) (all BioLegend).

The stained cells were then washed twice and resuspended in PBS^−/−^ + 2% HI-FCS (Thermo Fisher Scientific) with an equal amount of 4% PFA (Sigma-Aldrich) for fixing and stored at 4°C, away from direct sunlight, to be ran on a flow cytometer within 2 days. Before running, samples were washed once more and resuspended in 0.3 ml of PBS^−/−^. For the analysis of isolated human cells, data were acquired on the LSRFortessa (five lasers; BD Bioscience) using BD FACSDiva software with analysis and visualization of the data performed using FCS Express 7 (De Novo Software). Stained human whole-blood samples were analyzed on the Attune NxT Flow Cytometer (Thermo Fisher Scientific), all within the QMRI Flow Cytometry and Cell Sorting Facility.

### Macrophage culture and treatment

Isolated human monocytes were plated at 8 × 10^5^ per well into 24-well tissue culture plates (Sigma-Aldrich) and cultured in Iscove’s modified Dulbecco’s medium (IMDM; Thermo Fisher Scientific) supplemented with 10% heat-inactivated fetal calf serum (FCS; Thermo Fisher Scientific), 1% penicillin-streptomycin (Thermo Fisher Scientific), 1% l-glutamine (Thermo Fisher Scientific), and human recombinant M-CSF (100 ng/ml; PeproTech) to induce differentiation. Cells were cultured for 7 days at 37°C with fresh media applied at day 5. On day 7, cell detachment was achieved first by washing wells vigorously twice in warm PBS and then applying 500 μl of ice-cold PBS + 1% bovine serum albumin (BSA; Sigma-Aldrich) + 3 mM EDTA (Thermo Fisher Scientific) and leaving on ice for 25 min. Fully differentiated MDMs were plated overnight at 0.25 × 10^6^ per well on a 48-well culture plate in complete IMDM. LPS challenge was carried out with *P. aeruginosa* 10 LPS (100 ng/ml; Sigma-Aldrich) for 24 hours.

For in vitro culture of macrophages with CFTR modulators, monocytes were plated following negative selection from patient blood in IMDM + M-CSF (100 ng/ml) containing 3 μM VX-445 (aka elexacaftor), 5 μM VX-661 (aka tezacaftor), and 3 μM VX-770 (aka ivacaftor) (all modulators given together form the Kaftrio therapy and were provided by Vertex Pharmaceuticals). Modulators were all reconstituted to 10 mM stock in DMSO (Sigma-Aldrich) and then further diluted to a working concentration in IMDM to give a final concentration of DMSO in each well of 0.11% in the Kaftrio-treated wells. Vehicle control wells were also set up with the same concentration of DMSO minus the drug. Given the unknowns regarding stability and half-life of these new drugs, medium changes were carried out on days 3, 5, and 7 of the MDM culture. LPS treatments were carried out in the presence of the same concentrations of modulators and vehicle control.

### RNA extraction

Cells were lysed in the well for gene expression analysis using RLT buffer (QIAGEN) with 1% β-mercaptoethanol (Sigma-Aldrich) as a reducing agent to denature ribonucleases (RNases) and immediately stored at −80°C. RNA extraction was performed using the RNeasy Mini Kit (QIAGEN) with on-column deoxyribonuclease (DNase) I digestion also carried out before final elution using the RNase-Free DNase Set (QIAGEN), both as per the manufacturer’s instructions.

Quality and concentration of the RNA were assessed using LabChip GX Touch Nucleic Acid Analyzer (PerkinElmer), standard sensitivity assay, which generates a concentration (in ng/μl) and an RNA quality score (RQS) ranging from 1 to 10 calculated from the ratio of 28*S* to 18*S* ribosomal RNA (rRNA). Only samples with an RQS ≥ 9 (equating to all samples attempted) were used for CAGE sequencing. For RNA samples to be analyzed only by qRT-PCR, RNA concentration was determined using the NanoDrop 1000 Spectrophotometer (Thermo Fisher Scientific).

### Quantitative reverse transcription polymerase chain reaction

cDNA was synthesized from RNA for qRT-PCR. Sample concentrations were normalized to the lowest concentration sufficient to give a minimum of 300 ng of RNA by diluting with RNase-free water. cDNA was produced using a master mix containing reverse transcriptase and other reagents from the High-Capacity cDNA Reverse Transcription Kit (Thermo Fisher Scientific) and RNase Inhibitor (Thermo Fisher Scientific), according to the manufacturer’s instructions. Samples were incubated with the master mix in a thermal cycler for the following run cycle: step 1, 25°C for 10 min; step 2, 37°C for 120 min; step 3, 85°C for 5 min; and step 4, hold at 4°C. The cDNA product was then stored at −20°C. Primer sequences were designed using Primer3 and Ensembl web interfaces, NetPrimer (Premier Biosoft) and National Center for Biotechnology Information primer-BLAST to assess specificity. To minimize amplification of genomic DNA, primers were designed to either bind across exon-exon junctions or either side of a large intron (>2000 bp). Custom DNA oligos were purchased from Integrated DNA Technologies, unless stated. Sequences of primers used in this study are as follows: 18*S* (internal control) (forward, 5′-GTAACCCGTTGAACCCCATT-3′ and reverse, 5′-CCATCCAATCGGTAGTAGCG-3′), *MXI* (forward, 5′-GGCTGTTTACCAGACTCCGACA-3′ and reverse, 5′-CACAAAGCCTGGCAGCTCTCTA-3′; OriGene), *IFI44L* (forward, 5′-TGCACTGAGGCAGATGCTGCG-3′ and reverse, 5′-TCATTGCGGCACACCAGTACAG-3′; OriGene), *IFIT3* (forward, 5′-CCTGGAATGCTTACGGCAAGCT-3′ and reverse, 5′-GAGCATCTGAGAGTCTGCCCAA-3′; OriGene), and *OAS3* (forward, 5′-GAATTTCTCCAGCCCAACCG-3′ and reverse, 5′-GAAGAGCCACCCTTGACCA-3′). cDNA was first diluted 1:4 in RNase-free water. qRT-PCR was performed using the SsoAdvanced Universal SYBR Green Supermix kit (Bio-Rad) in MicroAmp Optical 96-Well Reaction Plates (48 and 96 wells; Thermo Fisher Scientific) and run on the Applied Biosystems StepOnePlus Real-Time PCR System (Thermo Fisher Scientific) with the following settings: holding stage, 95°C for 10 min; cycling stage, 95°C for 15 s and 60°C (this value was decided for each primer pair individually on the basis of optimal specificity as adjudged by analysis of the melt curve) for 1 min; melt curve stage, 95°C for 15 s, 57°C (this value was always chosen as the amplification temperature: 3°C) for 1 min, and 95°C for 15 s. CT values were obtained for each sample, and expression fold change was measured using the 2^−ΔΔCt^ method with normalization against 18*S* rRNA.

### CAGE library preparation and sequencing

CAGE libraries were prepared as described by Takahashi *et al.* (*25*) using 5 μg of total RNA as input material per sample. Samples were individually barcoded using the sequences outlined by Takahashi *et al.* (*25*) with each pooled library containing eight unique barcoded samples (the following barcodes were used: CTT, GAT, ACG, ATG, TAG, TGG, GTA, and GCC). The pooled libraries were quantified using the Qubit 2.0 Fluorometer (Thermo Fisher Scientific) with the Qubit dsDNA HS Assay Kit and assessed for quality and fragment size on the Agilent 2100 Electrophoresis Bioanalyser Instrument (Agilent Technologies) with the DNA HS Kit before being sent for sequencing on a high-throughput run on the Illumina HiSeq 2500 System (Illumina) at the Centre for Genomics Research, University of Liverpool.

### Data preprocessing and genome alignment

Quality control checks of the raw sequence data were first performed by FastQC (v0.11.7) (*76*) analysis of the eight FastQ files with summary and visualization produced using the MultiQC program (*77*) to assess tag sequence length, quality, and predicted adapter content. Following QC, the reads within each library were then separated by the presence of the unique barcodes listed above. Cutadapt (v1.16) (*78*) was then used to trim reads by removal of adapter sequences from the 5′ and 3′ ends before alignment of the trimmed tags to the human genome (hg38; release date: December 2013; downloaded from UCSC Genome Browser in January 2020; http://hgdownload.soe.ucsc.edu/goldenPath/hg38/bigZips/hg38.fa.gz) using Bowtie (v1.1.2) (*79*). Mapping files, produced here in SAM format, were then converted to BAM files using SAMtools (v1.9) (*80*). A further FastQC check was carried out, as before, at this point to assess trimmed read quality and the effectiveness of adapter removal in the trimmed tags.

### CTSS clustering, tag normalization, and differential expression analysis

CAGE Transcriptional Start Site (CTSS) files containing the starting genomic position of all CAGE tags with the number of reads for each tag were then created from BAM files using the genomecov command from BEDtools (v.2.26.0) (*81*). CTSS files were then converted to BED file format ahead of conversion to BigWig using bedGraphtoBigWig (v4) for use with the CAGEfightR analysis framework. Analysis of CAGE data was carried out using the CAGEfightR package (v1.5.1) as described by Thodberg and Sandelin (*82*, *83*), which uses CTSSs stored in BigWig files as input and clusters tags at a CTSS, promoter, and gene level to allow for analysis of differential gene expression, promoter usage, and enhancer activity, etc. Normalization of CTSS counts was calculated to TPM (number of tags observed at a given CTSS/total number of mapped tags × 1 million) before TPM values were summed across all samples to give a pooled CTSS signal using calcTPM and calcPooled commands. Only TSSs expressed at >1 TPM in more than six samples were included to exclude very lowly expressed, biologically irrelevant clusters. Differential expression was compared at a TSS and gene level with statistical analyses performed using the general linear model quasi-likelihood framework approach, with defined batch covariates (relating to each individual library) included as blocking factors.

### Gene set and TFBS enrichment analysis

Differential gene expression between defined experimental groups was calculated using CAGEfightR and the Limma-Voom package. The most highly enriched or depleted GO terms were determined using the goana and topGO functions in Limma with default setting to allow for easier interpretation of gene groups and biological process that were differentially expressed between groups. The most highly differentially expressed genes between groups were determined on the basis of a combination of logFC and *P*_ADJ_ and used to investigate enrichment of protein-protein interactions between groups using the STRING database with disconnected nodes in the network hidden but otherwise default settings (*84*).

For analysis of TFBS enrichment, the TSSs that were most differentially expressed between experimental groups were compared by extracting the candidate DNA binding motifs for each TSS, defined as being within 500 bp either side of the TSS peak. TFBSTools (*85*) and motifmatchr (*86*) packages were then used to identify motifs and test the prevalence of specific TFBSs within the most differentially expressed promoter against the prevalence that would be expected to occur at random.

### Gene editing the F508del mutation in iPSCs

CRISPR single gRNAs (sgRNAs) targeting the exon 11 of the F508del *CFTR* were designed for the targeted knock-in of CTT. The designed sgRNAs were first screened in silico for off-target activity using the COSMID webtool (*87*) to ensure high editing specificity with a low number of off-target sites with homologous sequences. An ssODN donor template complementary to the nontarget strand was designed (table S5) to correct the F508del in *CFTR* via the HDR of the DNA double-stranded break induced by Cas9 cutting. The ssODN donor was designed with homology around the cut site such that the CTT insertion was centered on it and contained four silent mutations upstream of the cut site to prevent Cas9 recutting of the inserted donor sequence. The sgRNA (purchased from Synthego with 2′-*O*-methyl modifications) was complexed with Cas9 protein and delivered as a ribonucleoprotein mixed with ssODN donor template to the iPSCs using optimized nucleofection parameters. The editing efficiency was assessed using the ICE webtool from Synthego.

### iPSC differentiation to macrophages

The differentiation of the iPSCs to macrophages was adapted from previously published protocols (*56*). Non-CF iPSCs were purchased from the stem cell core at Baylor College of Medicine, Human Stem Cell Core. CF iPSCs were a gift from the Rajagopal Lab at Harvard University. The iPSCs were stained for the pluripotency markers before differentiations to ensure differentiation potential of these cells. The iPSCs were detached using Accutase (Innovative Cell Technologies) and seeded into AggreWell 800 plates (STEMCELL Technologies) to form embryoid bodies (EBs). The EBs were maintained in the EB Formation Media (STEMCELL Technologies) for 4 days supplemented with vascular endothelial growth factor (50 ng/ml; PeproTech), bone morphogenetic protein 4 (50 ng/ml; PeproTech), and SCF (20 ng/ml; PeproTech) with 1 mM of Y-27632 (STEMCELL Technologies). On day 4, the EBs were harvested and seeded into six-well plates with 10 EBs per well and maintained in X-VIVO15 medium (Lonza) supplemented with M-CSF (100 ng/ml; R&D Systems), IL-3 (25 ng/ml; R&D Systems), 2 mM GlutaMAX (Invitrogen), 0.055 β-mercaptoethanol (Invitrogen), penicillin (100 U/ml), and streptomycin (100 μg/ml; Invitrogen). Monocytes were harvested from the media after 2 to 3 weeks of culture and plated for differentiation to macrophages. The X-VIVO15 medium was supplemented with M-CSF (100 ng/ml) for 5 to 7 days for full differentiation of the monocytes to macrophages. The macrophages were then activated with LPS (100 ng/ml; Sigma-Aldrich) for 24 hours before harvesting for downstream experiments and characterizations (fig. S4). The macrophages were stained with 1:50 human anti-CD14 (17000-1-AP, Thermo Fisher Scientific) and 1:200 anti-CD68 antibodies (R&D Systems, MAB20401) in confocal microscopy compatible eight-well Nunc Lab-Tek chamber slides (MilliporeSigma). The antibodies were titrated with isotype rabbit immunoglobulin G (IgG; 171870, Abcam) and mouse IgG2B (MAB004, R&D Systems). The cells were fixed using Fixation/Permeabilization Kit (554714, BD Biosciences) according to the manufacturer’s recommended procedure before blocking with 1% BSA in PBS with Tween 20 (PBST) at 4°C overnight. The next day, 5 μl of Human TruStain FcX (BioLegend) to block Fc receptors for 10 min at room temperature. Then, the primary antibody was incubated with cells for 3 hours; secondary antibodies were incubated for 45 min and Hoechst (Thermo Fisher Scientific)–stained (6 μg/ml) for 10 min at room temperature with washes with the BD Biosciences Perm/Wash kit (554723) in between.

## Notes

View/request a protocol for this paper from *Bio-protocol*.
