# Supplementary material for: CAGE sequencing reveals CFTR-dependent dysregulation of type I IFN signaling in activated cystic fibrosis macrophages
Source: Sci Adv. 2023 May 26;9(21):eadg5128. doi: 10.1126/sciadv.adg5128 (PMC10219589; doi:10.1126/sciadv.adg5128)
Supplement: Supplementary file 1 — Figs. S1 to S4 Tables S1 and S5 Legends for tables S2 to S4 [file sciadv.adg5128_sm.pdf]

Science Advances
AAAS

Supplementary Materials for

CAGE sequencing reveals CFTR-dependent dysregulation of type I IFN signaling in activated cystic fibrosis macrophages

Jonathan L. Gillan *et al.*

Corresponding author: Robert D. Gray, r.d.gray@ed.ac.uk

Sci. Adv. **9**, eadg5128 (2023)
DOI: 10.1126/sciadv.adg5128

The PDF file includes:

Figs. S1 to S4
Tables S1 and S5
Legends for tables S2 to S4

Other Supplementary Material for this manuscript includes the following:

Tables S2 to S4

Supplementary Information

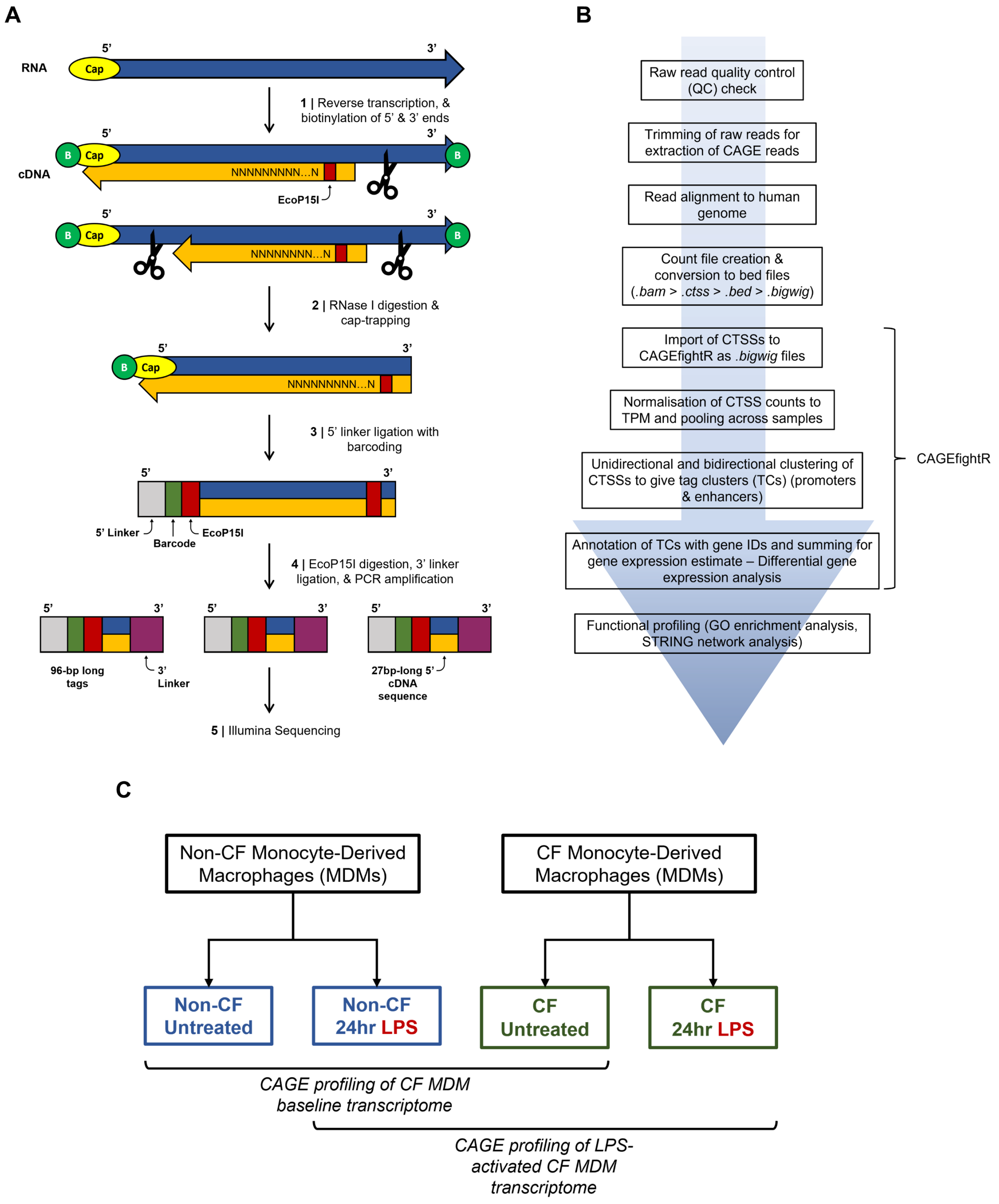

Supplementary Figure 1 | **Cap analysis of gene expression and experimental setup**. **A**. Schematic workflow of the process of preparing CAGE tags for Illumina sequencing from a single mRNA transcript. Scissors denote RNase digestion of single-stranded RNA. **B**. Summary of the CAGE library analysis workflow. **C**. Schematic of CAGE Experimental Design. Analyses were performed to determine the relative transcriptional activity of CF MDMs, both at a basal state and following LPS treatment.

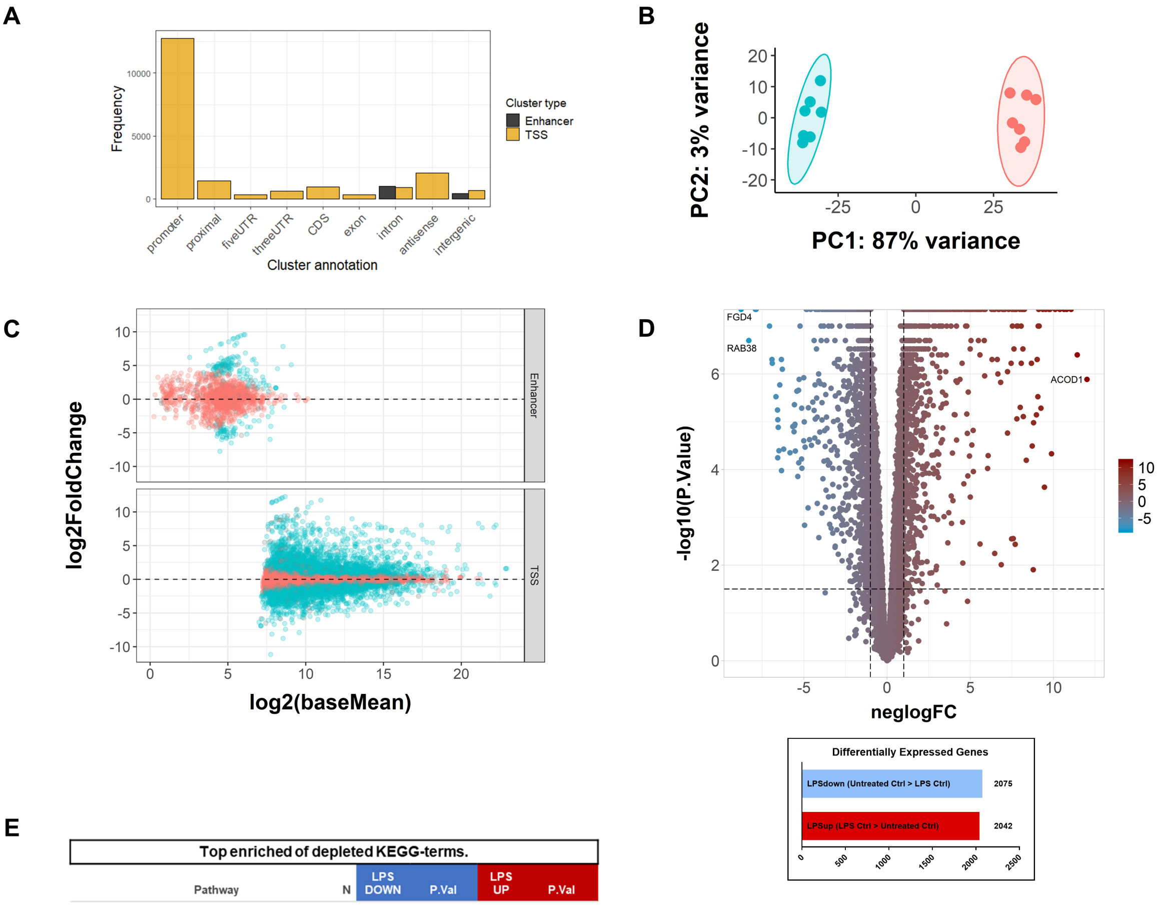

E

Top enriched of depleted KEGG-terms.						
	Pathway	N	LPS DOWN	P.Val	LPS UP	P.Val
path:hsa04060	Cytokine-cytokine receptor interaction	103	15	0.9440980	57	0.0000000
path:hsa01100	Metabolic pathways	940	266	0.0000000	181	0.6450943
path:hsa04061	Viral protein interaction with cytokine and cytokine receptor	42	3	0.9959922	27	0.0000000
path:hsa04668	TNF signalling pathway	84	14	0.8252925	40	0.0000000
path:hsa05169	Epstein-Barr virus infection	135	18	0.9891529	55	0.0000000
path:hsa05417	Lipid and atherosclerosis	148	27	0.7811088	57	0.0000002
path:hsa04064	NF-kappa B signaling pathway	73	14	0.6438959	33	0.0000009
path:hsa05140	Leishmaniasis	50	7	0.9390079	26	0.0000017
path:hsa04621	NOD-like receptor signaling pathway	121	15	0.9934814	47	0.0000018
path:hsa04657	IL-17 signaling pathway	62	7	0.9815268	29	0.0000022

F

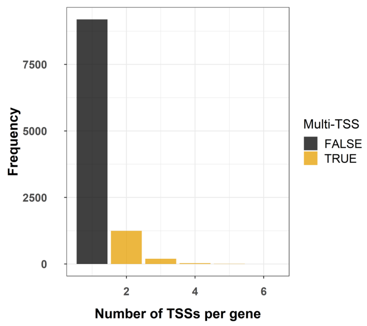

Top Genes Showing Differential TSS Usage				
	GeneID	Gene	# of Exons	FDR
1	8522	*GAS7*	3	0
2	10783	*NEK6*	4	0
3	3985	*LIMK2*	2	0
4	64759	*TNS3*	3	0
5	8837	*CFLAR*	3	0
6	11214	*AKAP13*	5	0
7	56949	*XAB2*	2	0
8	100423004	*MIR4315-1*	3	0
9	259307	*IL4I1*	2	0
10	8685	***MARCO***	2	0

G

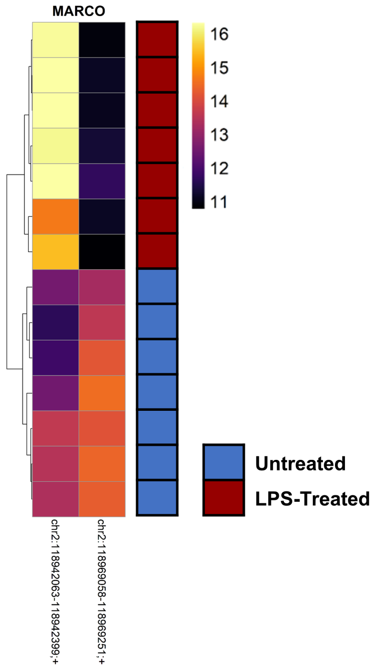

Supplementary Figure 2 | **CAGE profiling of *P. aeruginosa* LPS-activated human MDMs.** **A**. Uni- and bi-directional tag clusters in this set, showing total number of hits in clusters of each annotation category. **B**. Principal component analysis based on CTSS expression across all samples (each dot represents a single sample - % variance shown on each axis title). **C**. MA plot of differential uni- and bi-directional cluster expression between untreated non-CF and untreated CF macrophages. Blue points indicate a significantly differentially expressed gene with adjusted P.Value (PADJ) < 0.05. Grey points indicate a base expression level too low to enable DE analysis. **D**. Volcano plot displaying all detected genes (10,666) following aggregation and Entrez gene ID annotation of unidirectional tag clusters, by log fold change (logFC) and -log10P.Value. Dashed y intercept is at -log10P.Value of 1.5 (genes above this were considered statistically significant) with x intercepts at 1 and -1 logFC. The most highly significant and DE genes (logFC ≥ 9 or ≤ -7 and -log10P.Value ≥ 5) are labelled. Number of DE genes (PADJ < 0.05) between groups listed below the plot. **E**. Top enriched and depleted KEGG terms calculated from gene-level DE analysis. **F**. The number of TSSs detected in each gene and the top 10 genes (by FDR) showing significant differential promoter usage between untreated CF and non-CF groups. **G**. Heatmap displaying expression between untreated patient and control samples at active TSSs within the *MARCO* gene.

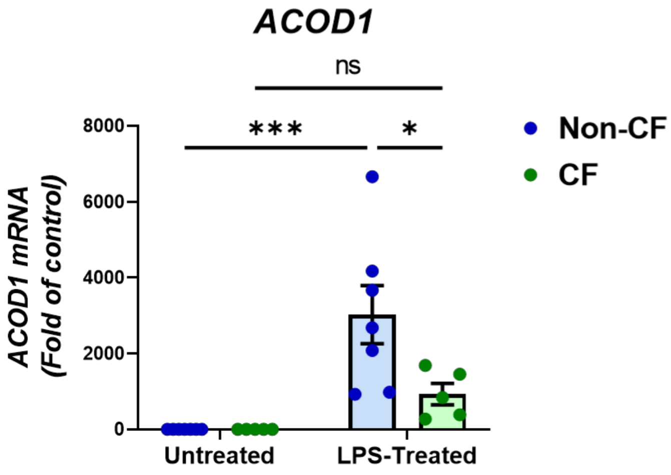

Supplementary Figure 3 | ***ACOD1* Expression in Healthy and CF CAGE Samples as Measured by qRT-PCR.** Gene expression was measured by qRT-PCR from cDNA synthesised from RNA isolates remaining from samples used for CAGE sequencing (5 of the 7 patient samples had sufficient RNA remaining to carry out this validation). CF and non-CF MDMs were stimulated with 100ng/ml *P. aeruginosa* LPS for 24 hours, as described. Expression values were calculated relative to *18S* expression using the $2^{-\Delta\Delta Ct}$ method and given as fold of untreated non-CF controls. * = P ≤ 0.05. Obtained via 2-way ANOVA with Tukey's multiple comparisons test. Data are presented as mean ± SEM.

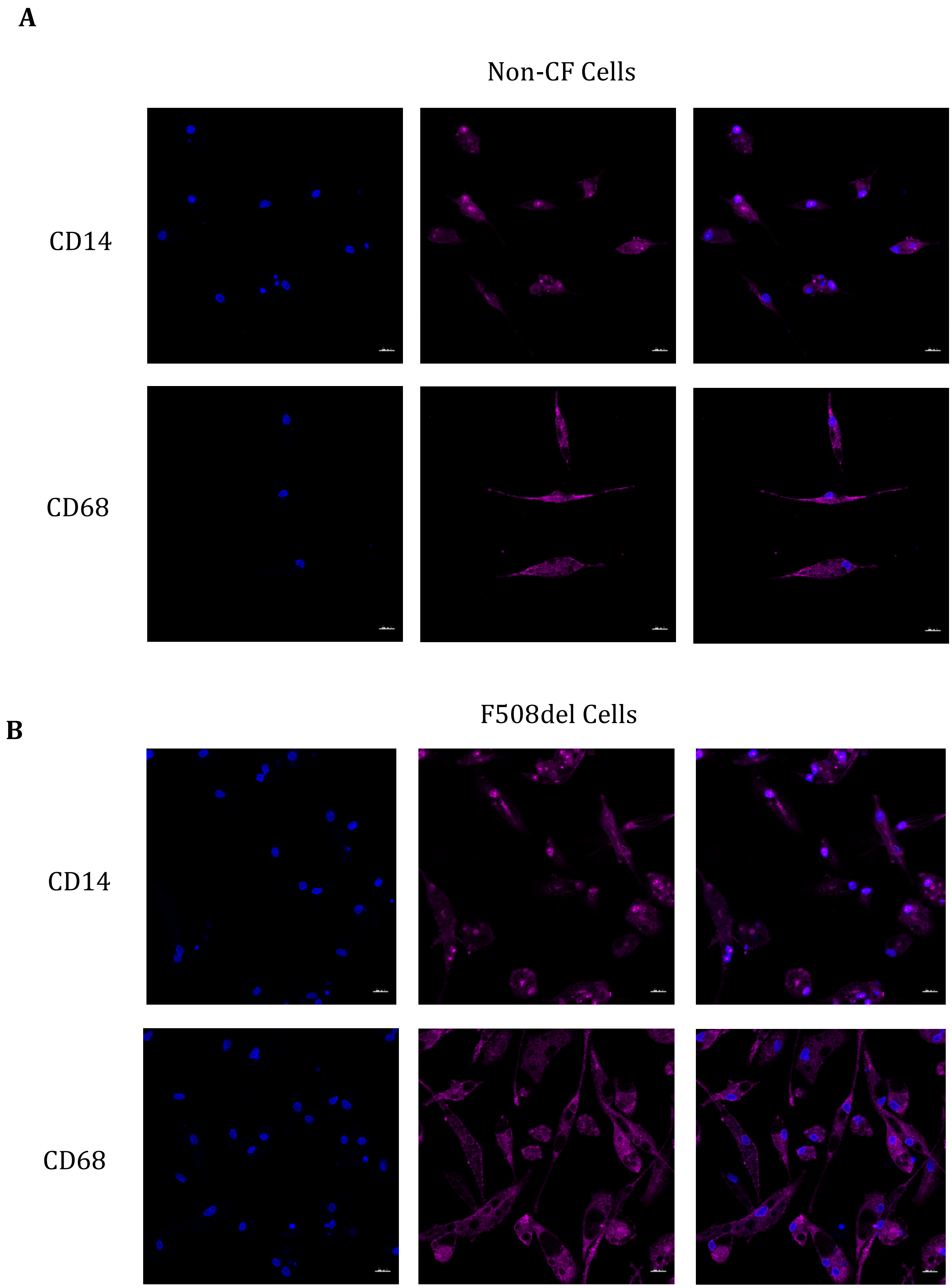
A
Non-CF Cells
CD14
CD68
B
F508del Cells
CD14
CD68

C

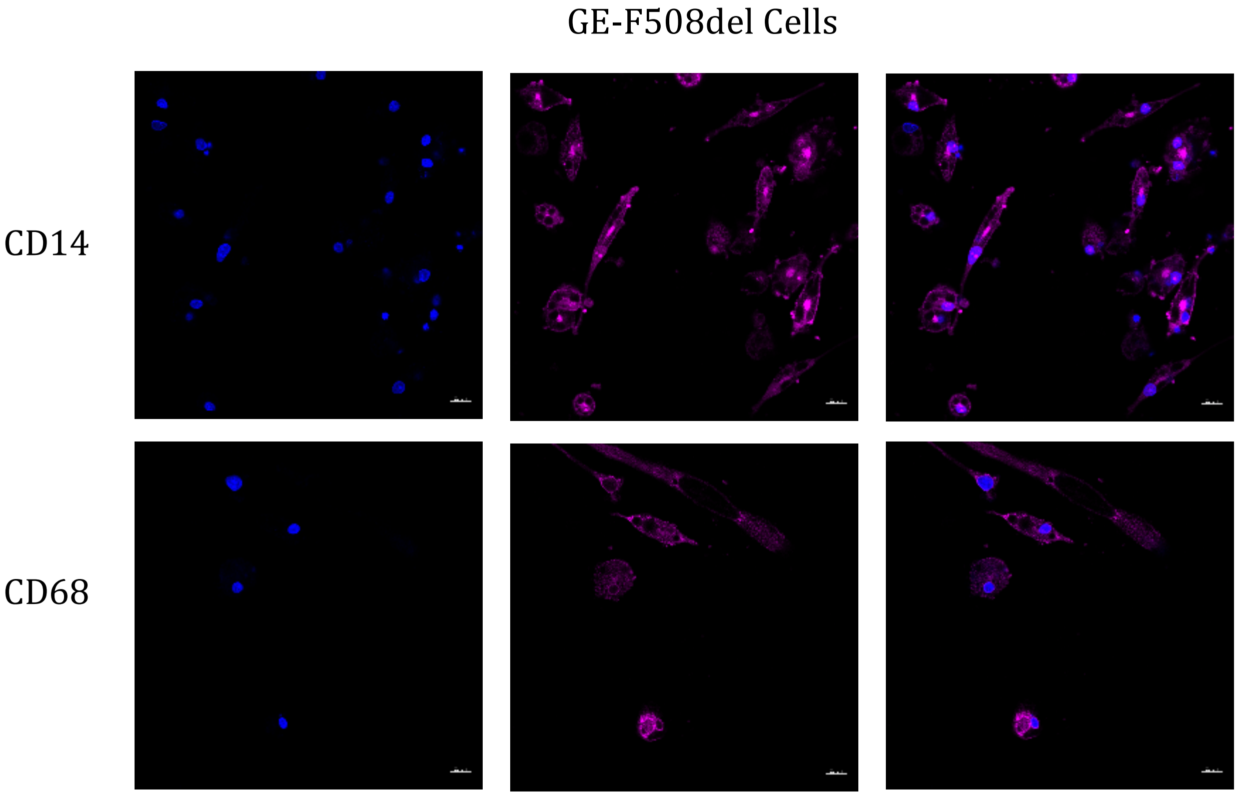

Supplementary Figure 4 | **CD14 and CD68 Staining of the iPS-Macrophages.** iPS macrophages stained after 24 hours of LPS stimualation stained positive for CD14 and CD68 for all three types of differentiated cells (non-CF, F508del, and GE-F508del). Scale bar: 20 μm.

	**Healthy Ctrls**	**CF**
n	7	7
**Age**	29.1 ± 6.6	32.3 ± 8.9
**Sex (% female)**	57%	29%
**CFTR mutation**		
**F508del/F508del**	71%	n/a
**F508del/Class I**	29%	n/a

Supplementary Table 1 | **Patient and Healthy Control Characteristics.** Continuous variable given as mean ± standard deviation.

Supplementary Table 2 | **Untreated Ctrl vs LPS Ctrl - TSS and Gene Level Differential Expression** (.xlsx)

Supplementary Table 3 | **Untreated Ctrl vs Untreated CF - TSS and Gene Level Differential Expression** (.xlsx)

Supplementary Table 4 | **LPS Ctrl vs LPS CF - TSS and Gene Level Differential Expression** (.xlsx)

Supplementary Table 5 | **gRNA and ssODN Donor Sequences**

	Sequence (5'-3')
**gRNA**	ACCATTAAAGAAAATATCAT
**ssODN Repair Template**	AAGAATTTCATTCTGTTCTCAGTTTTCCTGGATTATGCCTGGCACCATTAAGGAGAACATTATCTTTGGTGTTTCCTATGATGAATATAGATACAGAAGCGTCATCAAAGCATGCCAACTAGAAGAG
